# Pharmacokinetic analysis of new synthetic antimalarial N-251

**DOI:** 10.1186/s41182-019-0167-4

**Published:** 2019-07-05

**Authors:** Kazuaki Okada, Akira Sato, Akiko Hiramoto, Rena Isogawa, Yuji Kurosaki, Kazutaka Higaki, Shin-Ichi Miyoshi, Kyung-Soo Chang, Hye-Sook Kim

**Affiliations:** 1 Division of International Infectious Diseases Control, Faculty of Pharmaceutical Sciences, Okayama University, 1-1-1 Tsushima-Naka, Kita-Ku, Okayama, Okayama 700-8530 Japan; 2 Department of Biochemistry, Faculty of Pharmaceutical Sciences, Tokyo University of Science, 2641 Yamazaki, Noda, Chiba, 278-8510 Japan; 3 Department of Pharmaceutical Formulation Design, Faculty of Pharmaceutical Sciences, Okayama University, 1-1-1 Tsushima-Naka, Kita-Ku, Okayama, Okayama 700-8530 Japan; 4 Department of Pharmaceutics, Faculty of Pharmaceutical Sciences, Okayama University, 1-1-1 Tsushima-Naka, Kita-Ku, Okayama, Okayama 700-8530 Japan; 5 Department of Sanitary Microbiology, Faculty of Pharmaceutical Sciences, Okayama University, 1-1-1 Tsushima-Naka, Kita-Ku, Okayama, Okayama 700-8530 Japan; 6 Department of Clinical Laboratory Science, College of Health Sciences, Catholic University of Pusan, Busan, 46252 Republic of Korea

**Keywords:** Synthetic endoperoxide, 6-(1,2,6,7-tetraoxaspiro [7.11] nonadec-4-yl)hexan-1-ol (N-251), Pharmacokinetic (PK) study, Bioavailability (F), Antimalarial medicine

## Abstract

**Background:**

With the emergence and growing number of drug-resistant *Plasmodium falciparum*, a new drug for malaria control must be urgently developed. The new antimalarial synthetic compound N-251 was recently discovered. As an endoperoxide structure in the body, the compound shows high antimalarial activity and curative effects. We performed a pharmacokinetic (PK) analysis of N-251 under various conditions using mice to understand the inhibitory effect of N-251 in parasite-infected mice.

**Results:**

PK study of N-251 after intravenous and oral administration in mice showed plasma concentration of N-251 was decreased drastically by intravenous route. *C*_max_ was reached in 2 h after oral administration of N-251, and the level decreased to a level similar to that obtained after intravenous administration. The area under the curves (AUCs) of the plasma concentration of N-251 increased dose-proportionally in both administrations, and bioavailability (*F*) was approximately 23%. Additionally, *T*_max_, *C*_max_, AUC, and *F* increased in fasted mice compared to normal-fed mice after the administration of N-251, indicating the influence of diet on the absorption kinetics of N-251. Furthermore, in parasite-infected fasted mice, the plasma concentration-time profile of N-251 was similar to that in normal-fasted mice. Based on the PK parameters of single oral administration of N-251, we investigated the effect of multiple oral doses of N-251 (68 mg/kg three times per day for 2 days) in normal-fed mice. The plasma concentration of N-251 was between 10 and 1000 ng/mL. The simulation curve calculated based on the PK parameters obtained from the single-dose study well described the plasma concentrations after multiple oral dosing, indicating that N-251 did not accumulate in the mice. Multiple oral administrations of N-251 in mice were required to completely eliminate parasites without accumulation of N-251.

**Conclusions:**

N-251 has been selected as a potent antimalarial candidate. We found that N-251 showed short half-life in plasma, and AUCs increased proportionally to dose. With multiple doses of N-251, the plasma level of N-251 was greater than 10 ng/mL in normal-fed mice, and accumulation of N-251 was not observed; however, multiple treatments with N-251 are required for the complete cure of parasite-infected mice. Determining the appropriate dosage was an important step in the clinical applications of N-251.

## Background

Malaria is a serious infectious disease caused by *Plasmodium* parasites mainly in tropical and subtropical regions. More than 219 million individuals have been infected with malaria parasites, and 435,000 deaths have been reported (WHO World Malaria Report 2018). Unfortunately, the resistant of *Plasmodium falciparum* to commonly used antimalarials is widespread [1, 2]. Since 1979, artemisinin has been isolated from the Chinese herb *Artemisia annua* and has been used to treat *P. falciparum* malaria [3, 4]. Currently, artemisinin-based combination therapies (ACT) are recommended by the WHO as first-line treatments of *P. falciparum* malaria; however, the emergence of parasites resistant to ACT in the area near the Thai-Cambodian border has been reported [5–8]. This confirms the need for the urgent development of new antimalarial drugs that are effective against such resistant parasites.

**Fig. 1:**
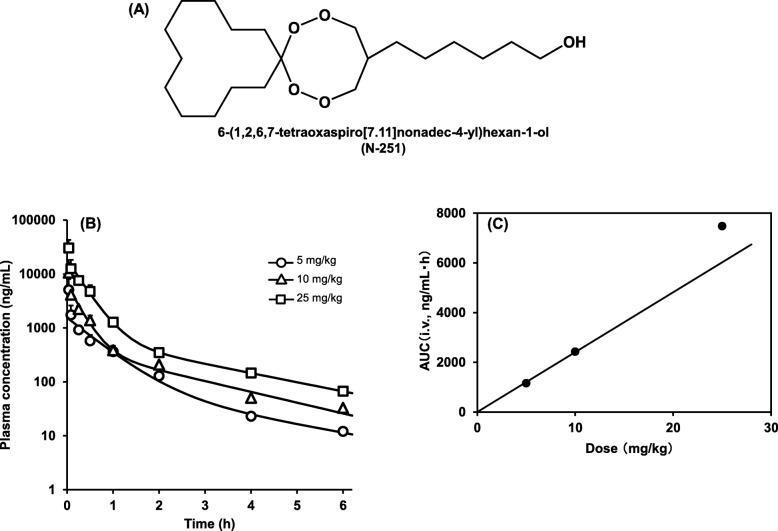
Structure of N-251 (**a**) and dose dependency of the pharmacokinetics of N-251 after intravenous in mice (**b**) and the correlation between dose and AUC_i.v_ (**c**). Results were expressed as mean with the bar showing the SD value. The solid lines are theoretical lines obtained in the fitting study

The endoperoxide structure of artemisinin is unique and is known to exhibit antimalarial activity [9]. In 2001, we synthesized several endoperoxide derivatives [10, 11]. We found that 6-(1,2,6,7-tetraoxaspiro [7.11] nonadec-4-yl)hexan-1-ol (N-251) (Fig. 1a) had strong antimalarial activity against *P. falciparum* in human erythrocytes cultured in vitro and *P. berghei* in mice in vivo [12]. In the analysis of antimalarial mechanisms of N-251, *P. falciparum* endoreticulum-resident calcium-binding protein (P*f*ERC) was found to be a possible candidate of the compound [13] that specifically inhibits trophozoite parasites [14]. Although the functions of P*f*ERC are not yet known as its location is believed to be localized in the endoplasmic reticulum [15], this protein may have an important role in the development of parasites.

Pharmacokinetics in animal studies is required to forward the development of N-251, which is a drug that can be of practical use. Although we have already started the additional study about the improvement of the bioavailability (*F*) of N-251 in non-infected rats [16], a pharmacokinetic (PK) analysis was performed under various conditions in mice, which included dose dependency, influence of diet, parasite infection, and number of administrations. In addition, the appropriate administration schedule for achieving the complete curative effect of N-251 against *P. berghei* in mice has already been determined [12]. By determining the PK parameters in this study, the appropriate administration schedules of N-251 for achieving drug efficacy without causing side effects can be confirmed.

## Methods

### Materials and preparation of N-251 solution

N-251 was synthesized, as described previously [12]. Cremophor® EL was purchased from Sigma Chemical Co. (St. Louis, MO, USA), and olive oil was obtained from Wako (Osaka, Japan). All other chemicals and reagents were analytical grade commercial products. Various doses of N-251 were administered as solutions to the groups of mice (six in each group) either orally (p.o., as solution in olive oil) or intravenously (i.v., as solution in 10% ethanol, 10% Chremophore® EL, and 80% saline (10:10:80 [*v*/*v*/*v*]).

### Animals

Male ICR mice (5 weeks old) weighing 25**–**30 g (Charles River Laboratories Japan, Yokohama, Japan) maintained at 25 °C and 55% humidity were allowed free access to standard laboratory chow (Clea Japan, Tokyo) and water. They were fasted for 24 h prior to and during the experiment but were allowed free access to water. Our study was carried out in accordance with the Guidelines for Laboratory Animals of Okayama University (OKU-2016150).

#### *P. berghei* parasites

*P. berghei* (NK65 strain) parasites were maintained by weekly passage of 1 × 10^6^ infected red blood cells in a healthy mouse via i.v. infusion, and parasite-infected mice were used for the rodent malaria parasites in the study.

### Preparation of plasma samples and analytical method

Blood samples were immediately centrifuged at 945*g* (4 °C, 10 min) to make plasma samples. To get N-251 from plasma samples, we used acetonitrile as solvent. A total of 300 μL acetonitrile was added to 100 μL plasma samples and vortexed for 10 s, and supernatant was obtained after centrifugation at 3259*g* (4 °C, 10 min). Quantitative analysis of N-251 was performed, as previously described [16]. Mouse plasma spiked with N-251 was prepared and analyzed using the same methods as standard curve of N-251.

### PK analysis after i.v. and p.o. administration of N-251 in fasted mice

We used healthy 5-week-old ICR male mice that were subjected to overnight fasting since the night a day before the test. To analyze N-251 after i.v. administration, doses of 25, 10, and 5 mg/kg were used for each group. The blood sample was collected at 2, 5, 15, and 30 min as well as 1, 2, 4, and 6 h post-dose, and a single blood sample was collected from a single mouse. Preparation of plasma samples of N-251 was described in the “Methods” section. Data were expressed as mean (*n* = 6) ± standard deviation (SD). Plasma concentration-time profiles after i.v. administration were fitted to a two-compartment model using the non-linear least-squares regression program MULTI [17]. Plasma concentration (Cp) was expressed using the following equation:

$$ \mathrm{Cp}=A\cdot EXP\left(-\alpha \cdot t\right)+B\cdot EXP\left(-\beta \cdot t\right). $$

where A and B are D ⋅ (α − k_21_)/Vd_1_/(*α* − β) and D ⋅ (k_21_ − β)/Vd_1_/(α − β), respectively. D, Vd_1_, and k_21_ are the dose, apparent distribution volume of central compartment, and distribution rate constant from the peripheral to central compartment, respectively, and *α* and β represent the slopes of *α* and β phases of log Cp, respectively. PK parameters were calculated as follows: AUC = *A*/*α* +B/β; total body clearance, CL_total_ =D/AUC; elimination rate constant, k_el_ = (Aβ + Bα)/(A + B); apparent distribution volume of peripheral compartment, Vd_2_ =k_12_/k_21_·Vd_1_; distribution volume at steady state, Vd_ss_ = Vd_1_ + Vd_2_; and mean residence time (MRT) = (k_12_+ k_21_)/(k_el_·k_21_).

To analyze the absorption kinetics of N-251, it was orally administered to fasted mice with dose of 210, 100, 68, 50, and 30 mg/kg. Blood sample was collected at 15 and 30 min as well as 1, 1.5, 2, 3, 4, and 6 h post-dose. Data were expressed as the mean (*n* = 6) ± SD. AUC and MRT after p.o. administration in fasting mice were calculated based on the trapezoidal rule. *F* was calculated with an AUC of 10 mg/kg of i.v. dose. The highest observed concentration was used as *C*_max_, and the time for *C*_max_ was defined as *T*_max_.

### Effects of food and parasite infection on the absorption behavior of N-251 after p.o. administration in mice

Normal-fasted and normal-fed mice were used for the analysis. Parasite-infected mice were prepared before the analysis, and a 0.61% infection rate in *P. berghei*-infected mice under fasting condition was used. The single p.o. dose of N-251 was 68 mg/kg. Blood sample was collected 15, 30, and 45 min as well as 1, 1.5, 2, 3, 4, and 6 h post-dose. Data were expressed as mean with the bar indicating the SD value.

### Effect of multiple p.o. dosing on the plasma concentration-time profile of N-251

N-251 was administered p.o. at a dose of 68 mg/kg every 8 h for 2 days in normal-fed mice. Blood sample was collected at 15, 30, and 45 min as well as 1, 1.5, 2, 3, 4, 6, 8, 10, 16, 18, 24, 26, 32, 34, 40, 42, 44, 46, and 48 h after administration. Data were expressed as mean (*n* = 6) with the bar indicating the SD value. Simulation of plasma concentration-time profile after multiple dosing was performed by utilizing the parameters obtained via fitting analysis for the plasma concentration-time curve after a single p.o. dose of N-251 based on the two-compartment model with the first-order absorption process. The equation and parameters used for the simulation were as follows:

$$ \mathrm{Cp}=X\cdot \mathrm{EXP}\left(- ka\cdot \left(t-{t}_{\mathrm{lag}}\right)\right)+Y\cdot EXP\left(-\alpha \cdot \left(t-{t}_{lag}\right)\right)+Z\cdot EXP\left(-\beta \cdot \left(t-{t}_{\mathrm{lag}}\right)\right), $$

where ka and *t*_lag_ are absorption rate constant and lag time, respectively, and X, Y, Z, α, and β are the hybrid parameters. Their values used for simulation were as follows: X = 8422 ng/mL/kg, ka = 0.845 h^−1^, Y = − 8419 ng/mL/kg, α = 1.09 h^−1^ ng/mL/kg, Z = 5.29 ng/mL/kg, β = 1.06 h^−1^, and lag time = 0.330.

## Results

### PK analysis after i.v. and p.o. administration of N-251 in fasted mice

The plasma concentration of N-251 after i.v. administration is shown in Fig. 1b, and each parameter is presented in Table 1. As shown in Fig. 1b, the plasma concentration of N-251 decreased in a time-dependent manner. The clearances of the 5 and 10 mg/kg groups were 4.30 and 4.11 L/h/kg, respectively, which had a similar degree. The clearance of the 25 mg/kg group was 3.35 L/h/kg. These data showed that the clearance is more likely to decrease with the increase in dose. However, the extent was not so large. By examining half-life, the drug may be immediately distributed from the central to the peripheral compartment as *T*_1/2*α*_ was 0.18–0.429 h and *T*_1/2*β*_ was 1.50–1.99 h (data not shown in Table 1). This result indicates that N-251 was rapidly eliminated from the plasma. The distribution volume was as large as 2.88–4.85 L/kg, which reflected that N-251 was a high fat-soluble compound. Figure 1 c shows the correlation between AUC_i.v._ and dose of N-251, indicating that the elimination of N-251 might be saturable at higher doses.

**Table 1:** Pharmacokinetic parameters of N-251 after intravenous administration in mice

Dose (mg/kg)	Pharmacokinetic parameters										
	AUC_iv_ (ng/mL·h)	CL_total_ (L/h/kg)	*k*_el_ (h^−1^)	Vd_1_ (L/kg)	Vd_2_ (L/kg)	Vd_ss_ (L/kg)	MRT_iv_ (h)	*A* (ng/mL)	*a* (h^−1^)	*B* (ng/mL)	*b* (h^−1^)
5	1161.50	4.30	1.33	3.23	1.62	4.85	1.12	1453.6 ± 465.9	1.62 ± 0.70	92.5 ± 182.0	0.35 ± 0.37
10	2433.64	4.11	2.60	1.58	2.35	3.93	0.96	5922.7 ± 1731.5	3.84 ± 1.23	410.0 ± 253.8	0.46 ± 0.15
25	7455.60	3.35	2.47	1.36	1.52	2.88	0.86	17,738.1 ± 1700.0	3.12 ± 0.55	690.4 ± 341.6	0.39 ± 0.10

Pharmacokinetic parameters were calculated using the equations described in the “Methods” section. *A*, *B*, *a*, and *b* are hybrid parameters in the equation describing the plasma concentration-time profile after intravenous administration, and they were expressed as SD

**Fig. 2:**
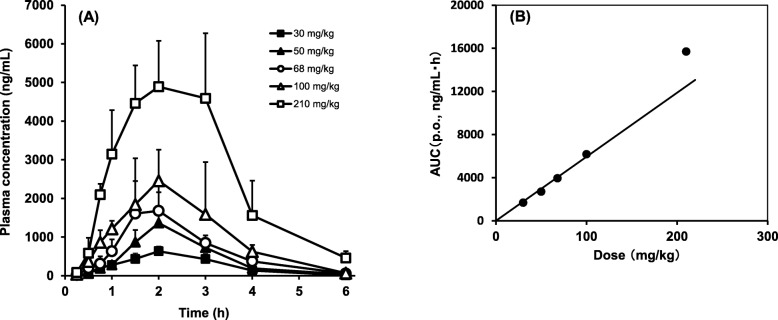
Dose dependency of the pharmacokinetics of N-251 after oral administration in mice (**a**) and correlation between dose and AUC_p.o_ (**b**). Results were expressed as mean with the bar showing the SD value. The solid lines are theoretical lines obtained in the fitting study

The graph of N-251 plasma concentration after p.o. administration was observed in Fig. 2a, and each parameter is presented in Table 2. To calculate for *F*, we referred to an AUC of 10 mg/kg of i.v. dose, which corresponded to the blood concentration range observed after p.o. administration. The *T*_max_ was 2 h in every group of dose. In Fig. 2a and b, both *C*_max_ and AUC_po_ had a liner correlation between 30 and 100 mg/kg, and *F* was approximately 0.22. However, the *F* value increased to 0.2982 at 210 mg/kg, indicating that the elimination process of N-251 would be saturated at high dose level.

**Table 2:** Pharmacokinetic parameters of N-251 after oral administration in mice

Dose (mg/kg)	Pharmacokinetic parameters				
	*C*_max_ (ng/mL)	*T*_max_ (h)	AUC_po_ (ng/mL·h)	*F*	MRT_po_ (h)
30	633.67 ± 128.70	2	1574.04	0.2156	2.45
50	1363.67 ± 798.98	2	2566.96	0.2110	2.49
68	1677.33 ± 811.23	2	3768.25	0.2277	2.46
100	2456.17 ± 802.04	2	5886.16	0.2419	2.46
210	4890.00±1188.24	2	15241.40	0.2982	2.74

*C*_max_ with SD and *T*_max_ are observed values. The AUC and MRT were calculated from 0 to infinity using the trapezoidal rule. *F* was calculated utilizing the AUC value of 2433.64 ng/mL·h, after intravenous administration of 10 mg/kg

### Effects of food and parasite infection on the absorption behavior of N-251 after p.o. administration in mice

**Fig. 3:**
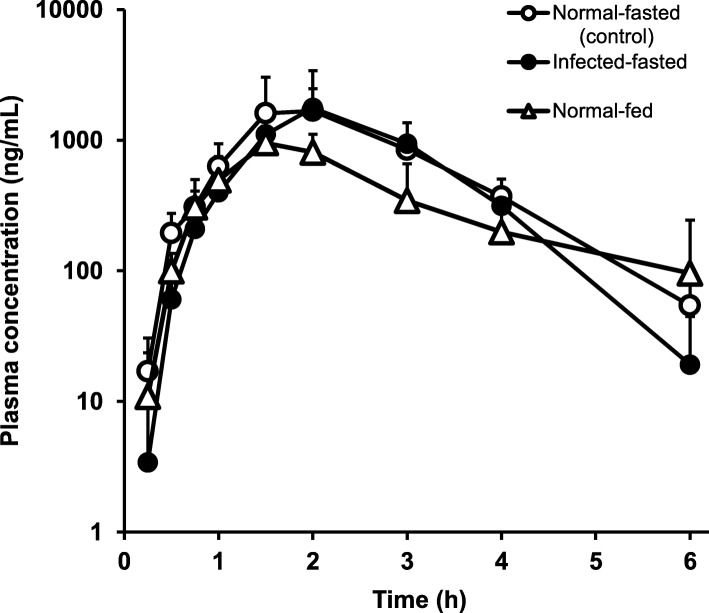
Effects of food and parasite infection on the absorption behavior of N-251. The dose of N-251 was 68 mg/kg. Results were expressed as mean with the bar showing the SD value

We examined the influence of diet on the p.o. absorption of N-251 at a single dose of 68 mg/kg in 0.61% parasite-infected mice. Figure 3 shows the results, and Table 3 depicts each of the obtained parameter. The plasma concentration of N-251 in normal-fasted and infected-fasted mice was quite similar, and the influence of parasite infection in mice was minimal. In contrast, the plasma concentration of N-251 in normal-fed mice was lower than that in normal-fasted mice, and the *T*_max_ increased at 1.5 h in normal-fed mice and 2 h in normal-fasted mice. The *C*_max_ also decreased in normal-fed mice (953 ng/mL) compared with normal-fasted mice (1677 ng/mL). The *F* values were also decreased in normal-fed mice compare with normal-fasted mice.

**Table 3:** Effect of food and parasite infection on the oral absorption of N-251 in mice

Conditions	Pharmacokinetic parameters				
	*C*_max_ (ng/mL)	*T*_max_ (h)	AUC_po_ (ng/mL·h)	*F*	MRT_po_ (h)
Normal-fasted (control)	1677.33 ± 811.23	2	3768.25	0.2277	2.46
Infected-fasted	1767.17 ± 1649.75	2	3273.35	0.1978	2.45
Normal-fed	952.50 ± 332.49	1.5	2282.17	0.1379	3.01

The *C*_max_ with SD and *T*_max_ were the observed values. AUC and MRT were calculated from 0 to infinity using the trapezoidal rule. *F* was calculated utilizing the AUC value of 2433.64 ng/mL·h after the intravenous administration of 10 mg/kg

### Effect of multiple p.o. dosing on plasma concentration-time profile of N-251

**Fig. 4:**
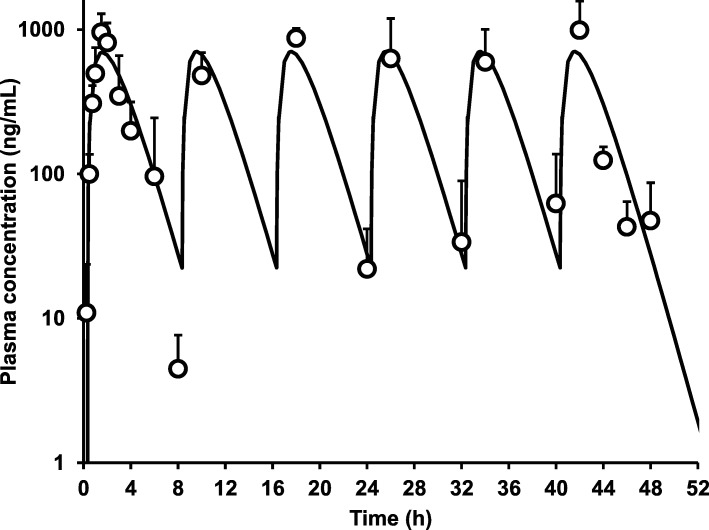
Effect of multiple oral dosing on plasma concentration-time profile of N-251. A dose of 68 mg/kg of N-251 was orally administered every 8 h in normal-fed mice. Results were expressed as mean with the bar showing the SD value. The solid line was the simulation line calculated utilizing the equation shown in the “Methods” section as obtained in the fitting study for plasma concentration profile after a single oral administration

We considered the plasma concentration that was maintained after the administration of 68 mg/kg p.o. of N-251 three times a day for 2 days in normal-fed mice. Figure 4 shows the measured values of plasma concentration after repeated administration (open circles). It was measured immediately before the administration and 2 h post-dose when the plasma concentration of N-251 was the lowest. The solid line represents the simulation curve calculated based on the absorption kinetics obtained after a single dose of N-251 at 68 mg/kg. Results showed that minimal plasma concentration was more than 10 ng/mL, except 8 h after the first administration. In addition, the maximal concentration of N-251 in mice did not increase during multiple dosing schedules, and the plasma profile was well described via a simple simulation based on the absorption kinetics obtained after a single p.o. dosing, indicating that multiple treatments of N-251 are safe for parasite-infected mice after p.o. treatment from the view point of pharmacokinetics. In our previous study, the EC_50_ value of N-251 *P. falciparum* (FCR-3 strain) in vitro was 8.6 ng/mL, which is equivalent to 2.3 × 10^−8^ M [12]. The plasma level of N-251 was between 10 and 1000 ng/mL, indicating it can eliminate parasites and has curative effects in mice.

## Discussion

The PK study of compounds for drug development of a drug used to treat malaria is an important step for further assessment in clinical trials. We discovered N-251 from random drug screening system for malaria in our university. N-251 and its related compounds had antimalarial activity and curative effects in *P. falciparum* in vitro and *P. berghei* in vivo in the mouse model in our previous reports [10–14]. Our antimalarial candidate N-251 has no UV absorption capability, and the standard HPLC system cannot be used. We must identify a new detection method using LC-MS/MS system. In 2015, we developed a detection method for N-251, and the first study of pharmacokinetics of N-251 formulation was reported [16]. N-251 was more lipophilic than griseofulvin (clogP = 2.88) [18] and the value of N-251 clogP was approximately 6.67. The solubility of N-251 in water was 7 μg/mL, which was similar to that of griseofulvin (5**–**6 μg/mL) [19]. In contrast, N-251 was dissolved in olive oil at 92 mg/mL and at 65 mg/mL in Cremophor® EL [16]. Therefore, we used olive oil in the p.o. solution solvent and Cremophor® EL-based i.v. solutions in the study.

In terms of the PK parameters of p.o. and i.v. N-251, N-251 has a short half-life, and toxicity can be minimized by discontinuing the administration of N-251. *F* in p.o. administration ranged from 21.1% to 29.8%. Based on the PK study of artemisinin-based drugs, it had a short half-life (below 1 h) and *F* was approximately 8–10% [20]. Although it cannot be directly compared due to specific differences, N-251 is assumed to migrate to the systemic circulation at the same rate and to have the same efficacy as an artemisinin-based drug. Thus, N-251 can be administered as an oral dosage form.

In the present study, it takes 2 h to reach the *C*_max_ after p.o. administration, and the *F* is almost constant ranging from 30 to 100 mg/kg. Total body clearance in i.v. administration of 5 and 10 mg/kg doses is quite similar. In 25 mg/kg i.v. administration and 210 mg/kg p.o. administration (the largest dose in each dosage), clearances were more likely to decrease compared to those in other doses, which may be due to the saturation of N-251 elimination when the plasma concentration is relatively high.

We compared PK data between normal-fasted *P. berghei*-infected mice and normal-fasted mice, with a dose fixed at 68 mg/kg. The plasma concentration of N-251 showed no differences between infected mice (infection rate of 0.61%) and healthy mice. A higher amount of N-251 was distributed to infected erythrocytes than healthy erythrocytes. The plasma concentration was not affected because only 0.61% of erythrocytes are infected. Approximately 5% of infected mice used as the model of severe malaria also showed similar results (data not shown). Based on these data, the PK data in healthy mice can be applied to the treatment design for infected mice. In terms of the influence of diet on pharmacokinetics, *T*_max_ was observed at an earlier time period (1.5 h), and the *C*_max_ decreased to 950 ng/mL, which was 57% of normal-fasted mice. The AUC decreased to about 60% in fed mice. This result suggests that some interaction between N-251 and diet suppressing the absorption might have occurred.

The PK analysis of repeated administration was based on the supposition of actual clinical experiments. We gained parameters based on the result of single p.o. administration in normal-fed mice. *C*_min_ was more than 10 ng/mL except 8 h after the first administration. The PK and pharmacodynamic (PD) parameters are important in therapeutic drug monitoring for the proper use of drugs, such as antibacterial and anticancer drugs [21]. For antibacterial drugs, the PK/PD parameters that are correlated to drug effects are classified into three: *C*_max_/minimum inhibitory concentration (MIC), AUC/MIC, and time above MIC. We applied this approach to N-251, and it was assumed that there was no difference in the drug effect of N-251 between i.v. and p.o. administration based on the result of a 4-day suppressive test (ED_50_ i.v. 22 mg/kg; p.o. 15 mg/kg) [11]. However, *F* in p.o. administration in this experiment was 21.1–29.8%, and the *C*_max_ and AUC were also lower in p.o. administration than in i.v. administration. Therefore, it was assumed that the drug effect did not correlate to either *C*_max_ or AUC. Elimination from the body between i.v. and p.o. administration with the short half-life of N-251 is less than 2 h, and it was quickly eliminated from the plasma. In repeated administration, *C*_min_ was more than 10 ng/mL except at 8 h after the first administration. Adequate plasma concentration was maintained after the second administration based on the EC_50_ value of N-251 at 8.6 ng/mL (= 2.3 × 10^−8^ M). In the case of a 68-mg/kg dose administered three times a day for 3 days, the plasma concentration was maintained at more than 10 ng/mL for 72 h. This mean effective blood concentration was maintained for at least three times in the life cycles of rodent malaria parasites. Therefore, we administered N-251 following the treatment schedule with this condition in infected mice. As shown previously [12], malaria parasites decreased after 16 h, and parasites were not observed in the bloodstream 36 h after treatment. No parasites were observed for 2 months after the last administration, and all mice were completely cured without recrudescence.

This research shows the PK data of N-251 via i.v. and p.o. administrations in mice, which is a cornerstone of the formulation and scaling up of research in the future. Furthermore, the drug effect is correlated to plasma concentration in N-251.

## Conclusion

We carried out a PK study for the new antimalarial candidate N-251 after multiple p.o. administrations. N-251 had a short half-life in the plasma, and multiple p.o. administrations are required in parasite-infected mice. Interestingly, the plasma level of N-251 is between 10 and 1000 ng/mL, and it does not accumulate in the mice. Data indicated that N-251 should be a safe drug for mammals, and a short half-life will require multiple treatments to completely eliminate the parasites without causing toxicity. This study first determined the PK parameters of N-251 to completely cure mice with malaria parasite infection.

## Data Availability

Not applicable

## Abbreviations

ACT: Artemisinin-based combination therapies
F: Bioavailability
i.v.: Intravenous
MIC: Minimum inhibitory concentration
N-251: 6-(1,2,6,7-tetraoxaspiro [7.11] nonadec-4-yl)hexan-1-ol
p.o.: Per oral
PD: Pharmacodynamic
PK: Pharmacokinetic
